# Impact of hormonal phases on salivary characteristics and oral hygiene in women: a cross-sectional comparative study

**DOI:** 10.1186/s12903-025-07284-5

**Published:** 2025-11-27

**Authors:** Ashwini S. Colaco, Aaptha Rai, Arun Mayya, Akshatha Chatra, Shashi Rashmi Acharya

**Affiliations:** 1 Department of Conservative Dentistry and Endodontics, A.J. Institute of Dental Sciences, Mangalore, 575004 Karnataka India; 2 Department of Conservative Dentistry and Endodontics, Manipal College of Dental Sciences, Manipal Academy of Higher Education, Manipal, 576104 Karnataka India

**Keywords:** Salivary flow rate, Salivary pH, Oral hygiene index (OHI-S), Menstrual cycle, Pregnancy, Menopause, Preventive dentistry, Women’s health

## Abstract

**Background:**

Hormonal fluctuations across a woman’s life stages can influence systemic and oral health. However, comparative data on salivary characteristics and oral hygiene across different hormonal phases remain limited. This study aimed to evaluate the salivary flow rate, salivary pH, and oral hygiene status among women in the menstruating, mid-cycle (ovulatory phase), pregnant-third trimester, and menopausal phases.

**Methods:**

In this cross-sectional study, 80 women (*n*=20 per group: mid-cycle (ovulatory phase), menstruating, pregnant-third trimester, menopausal) were examined. Unstimulated salivary flow (mL/5 min) and pH were measured; oral hygiene was assessed with the OHI-S. Outcomes were analyzed by ANCOVAwith age as a covariate. When omnibus effects were significant, Tukey-adjusted pairwise comparisons of estimated marginal means (EMMs) were performed. Effect sizes are reported as partial eta-squared (*η²p*) values; salivary flow is reported as mL/min for interpretability.

**Results:**

After adjusting for age, salivary flow differed by group (*F*=92.10, *p*<0.001; *η²p*=0.787). The adjusted means (mL/min) were as follows: mid-cycle 0.831, menstruating 0.912, pregnant 0.427, and menopausal 0.460; menstruating and mid-cycle were greater than pregnant and menopausal (all *p*<0.001), with no difference between pregnant and menopausal women (*p*=0.980). Salivary pH also differed (*F*=16.55, *p*<0.001, *η²p*=0.398): mid-cycle 7.65, menstruating 7.63, pregnant 6.93, menopausal 6.63; cycling groups exceeded pregnant and menopausal (most *p*<0.01), whereas mid-cycle vs menstruating and pregnant vs menopausal groups were not significant. Age was not associated with flow (*p*=0.931) or pH (*p*=0.843). Oral hygiene categories differed by group (Fisher’s exact *p*<0.001), with poorer hygiene in pregnant and menopausal women. However, the cross-sectional design precludes causal inference, findings are restricted to third-trimester pregnancies, and unmeasured factors such as oral hygiene, diet, socioeconomic status, and medication use may have influenced outcomes.

**Conclusions:**

The study indicates that hormonal phases, particularly third-trimester pregnancy and menopause, are associated with reduced salivary flow, lower pH, and poorer oral hygiene, underscoring the need for stage-specific preventive strategies and collaboration between gynecologists and dental professionals.

## Introduction

Women experience dynamic hormonal changes throughout their life stages, such as puberty, menstruation, pregnancy, and menopause, which significantly influence systemic and oral health [1, 2]. Hormonal fluctuations have complex effects on oral tissues; for example, elevated estrogen and progesterone levels around menses can increase gingival blood flow and inflammation (“menstruation gingivitis”) in susceptible women [3]. During pregnancy, profound hormonal changes have been linked to gingivitis, periodontitis, and other oral manifestations, including dental caries, pyogenic granuloma, and candidiasis, as highlighted in a recent systematic review [4]. Menopause, typically occurring in the middle-40 s to mid-50 s, is characterized by a decrease in estrogen that can lead to xerostomia (dry mouth) and a heightened risk of periodontal disease [5–7]. These hormonal phases can increase the susceptibility of women to oral infections and mucosal changes, potentially impairing their quality of life.

Saliva is an easily obtainable biofluid that is pivotal for maintaining oral homeostasis. It lubricates food, protects mucosal surfaces from microbial overgrowth, clears debris, buffers acids, and aids in the remineralization of teeth [8]. Saliva has been described as a “proactive gateway” to predict health and disease status [9]. Analysis of salivary parameters (flow rate, pH, buffering capacity, and composition) can thus provide valuable insights into an individual’s oral and systemic health. Previous studies have examined saliva under various conditions; however, the associations between the physicochemical properties of saliva and the different phases of the female life cycle remain underexplored. Some evidence suggests that salivary flow and pH may fluctuate with hormonal levels; for example, studies have examined menstruation alone [3], pregnancy [10, 11], or menopause in isolation [12–14]. However, few studies have directly compared these hormonal phases within a single design, and none, to our knowledge, have simultaneously evaluated menstruating, mid-cycle (ovulatory phase), pregnant, or postmenopausal women via the same methods. In light of these gaps, the present study aimed to evaluate and compare the salivary flow rate, pH, and oral hygiene status, as measured by the simplified oral hygiene index (OHI-S), among mid-cycle (ovulatory phase) women, menstruating women, pregnant women, and postmenopausal women. We hypothesized that pregnancy and menopause, in particular, would adversely affect salivary parameters and oral hygiene relative to women with regular hormonal cycles.

## Materials and methods

This cross-sectional observational study was conducted on women attending the departments of obstetrics and gynecology and outpatient dental clinics in a tertiary care hospital. Ethical clearance was obtained from the Institutional Ethics Committee (IEC/ENDO23/176/V1), and written informed consent was obtained from all participants. Reproductive age was defined as 18–45 years, whereas women above 45 years with cessation of menstruation for at least one year were categorized as post-menopausal, representing the nonreproductive age group. The participants were selected from four groups on the basis of their hormonal status: (1) healthy pregnant women in their third trimester who were receiving routine antenatal care; (2) Menstruating women in the reproductive age group during the first three days of their menstrual period with regular 28–30-day cycles; (3) menopausal women aged 45 years and above with a history of natural menopause (defined as ≥ 12 months of amenorrhea, consistent with WHO criteria); and (4) mid-cycle women in the reproductive age group with regular 28–30-day cycles who were not menstruating at the time of saliva collection. These women were recruited specifically during the mid-cycle phase (days 12–16), representing a hormonally stable period used as the reference control group. This classification aimed to isolate the effects of active hormonal fluctuations (as seen in menstruation and pregnancy), hormonal cessation (in menopause), and a hormonally stable reference state (mid-cycle control) on salivary and oral health parameters.

### Inclusion and exclusion criteria

 Only systemically healthy women who met the above group definitions and were within the typical range for each category (e.g., reproductive age for menstruating controls, ≥ 45 years for menopausal women) were included. The exclusion criteria were any systemic illnesses or conditions (e.g., diabetes) that could affect salivary flow or oral health; current or recent (past three months) use of antibiotics or any medications known to alter salivary output; signs or symptoms of xerostomia; and any ongoing dental infections or oral lesions requiring urgent care.

### Sample size calculation

 The required sample size was estimated using G*Power (v3.1.9.7) for ANCOVA, which compared four independent groups with age as a covariate. A minimum of 19 participants per group (total *N* = 76) were required to detect a large effect (Cohen’s *f* = 0.4) at α = 0.05 and power = 0.80. The large effect size follows Cohen’s conventions, and prior studies have suggested substantial group differences in salivary biomarkers across hormonal stages [15]; 20 participants per group (*N* = 80) were included to ensure adequate power.

### Sampling technique

 The required data were collected from women attending the Department of Obstetrics and Gynecology and outpatient dental clinics. A consecutive sampling technique was used to collect data from 20 subjects per group from subjects satisfying the inclusion and exclusion criteria.

### Oral health assessment

 A full-mouth oral examination was performed for each participant by a trained dental examiner. Oral hygiene status was evaluated using the simplified oral hygiene index (OHI-S) developed by Greene and Vermillion [16]. In addition, dentition status (DMFT) and periodontal parameters (bleeding on probing, pocket depth, and clinical attachment loss) were recorded according to WHO criteria. All examinations were performed by a single trained examiner who was calibrated prior to data collection under the supervision of a senior faculty member, ensuring consistency in OHI-S scoring. However, the OHI-S was considered the primary outcome for comparative analysis in this study. Any other oral findings, such as enamel fluorosis, dental erosion, dental trauma, or mucosal lesions, were documented, and the appropriate WHO oral health assessment codes/scores were assigned for each condition.

### Saliva collection and sialometric analysis

 Unstimulated whole saliva was collected from each participant to assess the salivary flow rate and pH. All samples were obtained between 9:00 AM and 12:00 noon to minimize diurnal variations. The participants were instructed to refrain from eating, drinking, or oral hygiene procedures for at least one hour before sample collection. Unstimulated saliva was chosen because it better reflects baseline glandular function under resting conditions, and previous work has shown that unstimulated samples retain key metabolic markers comparable to those in stimulated saliva in comparative salivary analyses [17]. Each subject was seated comfortably and given a few minutes to relax before collection. Saliva was collected by the spitting method: the participant was asked to pool saliva from the floor of the mouth and then spit into a graduated test tube via a funnel every 60 s for a total of 5 min. During collection, the subjects were advised to avoid swallowing or speaking. The total volume of saliva collected in 5 min was measured in milliliters, and the salivary flow rate (SFR) was calculated and expressed in mL per 5 min (later converted to mL/min for reporting). After flow measurement, salivary pH was immediately assessed by dipping a pH indicator strip into the sample. The color change on the pH strip was compared against a standard pH color chart to determine the pH value of the saliva to the nearest 0.2 unit.

#### Statistical analysis

The data were entered into Microsoft Excel and analyzed in Jamovi (v2.3.24). Continuous outcomes (salivary flow rate, salivary pH) were compared across four groups (mid-cycle, menstruating, pregnant-third trimester, menopausal) using analysis of covariance (ANCOVA) with age as a covariate. Prior to ANCOVA, the following assumptions were examined: normality of residuals (Shapiro–Wilk), homogeneity of variances (Levene’s test), and homogeneity of regression slopes (tested via Group×Age interaction). As the interaction terms were not significant, the parallel-slopes assumption was considered satisfied. Model effects are reported with *F*, *p*, and partial eta-squared (*η²p*) as the effect size. No influential outliers were detected in the salivary flow or pH data, and all values were retained for analysis. When the adjusted omnibus test was significant, pairwise comparisons of estimated marginal means (EMMs) were performed with Tukey adjustment. For interpretability, salivary flow is reported in mL/min (converted from raw 5-min volumes by dividing by five). Oral hygiene categories (OHI-S: good/fair/poor) were compared using Fisher’s exact test. Statistical significance was set at *p* < 0.05.

## Results

This study evaluated salivary parameters and oral hygiene scores across four groups of women: Mid-cycle (ovulatory phase), menstruating, pregnant-third trimester, and menopausal. Table 1 summarizes the age distribution of the women participants.The groups differed in age by design (menopausal markedly older); therefore, age was included as a covariate in all the models.

**Table 1:** Age distribution of the participants

Group	*n*	Mean	SD*
Mid-cycle Women	20	28.75	4.66
Menstruating Women	20	26.05	3.19
Pregnant Women (third trimester)	20	26.35	4.07
Menopausal Women	20	54.95	4.85

### Salivary flow rate (mL/min; age-adjusted)

The Group×Age interaction was nonsignificant (*p* = 0.080), supporting the homogeneity of the regression slopes. Levene’s test indicated no evidence of heteroscedasticity (*p* = 0.063). Shapiro–Wilk on residuals for salivary flow indicated mild deviation (*W* = 0.958, *p* = 0.010**).** Given equal group sizes and large *η²p* values, the ANCOVA results are considered robust; model residual diagnostics were otherwise acceptable.

ANCOVA revealed a strong group effect on salivary flow (*F* = 92.10, *p* < 0.001, *η²p* = 0.787), whereas age was not significant (*p* = 0.931). According to Cohen’s benchmarks, *η²p* ≥ 0.14 indicates a large effect. The age-adjusted EMMs (mL/min) were as follows: mid-cycle 0.831, menstruating 0.912, pregnant 0.427, and menopausal 0.460 (95% CIs in Table 2). Tukey post hoc tests on EMMs revealed that menstruating and mid-cycle > pregnant and menopausal (all *p* < 0.001); pregnant vs. menopausal was not significant (*p* = 0.980); and menstruating vs. mid-cycle was not significant (*p* = 0.080) (Table 3).

**Table 2:** Age-adjusted estimated marginal means (EMMs) for salivary flow and pH across study groups

	Salivary flow (mL/min)	Salivary pH
Group	EMM (SE) [95% CI]	EMM (SE) [95% CI]
Mid-cycle women	0.831 (0.027) [0.777–0.885]	7.646 (0.108) [7.432–7.860]
Menstruating women	0.912 (0.032) [0.849–0.975]	7.635 (0.126) [7.384–7.886]
Pregnant – third trimester	0.427 (0.031) [0.365–0.489]	6.932 (0.124) [6.686–7.179]
Menopausal women	0.460 (0.062) [0.336–0.584]	6.633 (0.247) [6.141–7.125]

Note: EMMs adjusted for age. *SE *standard error, *CI *confidence interval

Salivary flow converted from 5-min volume to mL/min

**Table 3:** Tukey-adjusted pairwise comparisons of salivary flow and pH across study groups

Comparison	Δ Salivary flow (mL/min)	*p* value	Cohen’s d	Δ Salivary pH	*p* value	Cohen’s d
Mid-cycle vs. Menstruating	−0.081	0.080	−0.79	0.011	0.999	0.03
Mid-cycle vs. Pregnant-third trimester	0.404	< 0.001	3.96	0.714	< 0.001	1.76
Mid-cycle vs. Menopausal	0.371	< 0.001	3.63	1.013	0.010	2.50
Menstruating vs. Pregnant-third trimester	0.485	< 0.001	4.75	0.703	< 0.001	1.73
Menstruating vs. Menopausal	0.452	< 0.001	4.42	1.002	0.023	2.47
Pregnant-third trimester vs. Menopausal	−0.033	0.980	−0.32	0.299	0.814	0.74

Note: Positive differences (Δ) indicate higher values in the first-named group. Cohen’s *d*, the effect size interpreted as small (d ≈ 0.2), medium (*d* ≈ 0.5) and large (*d* ≥ 0.8).

### Salivary pH (age-adjusted)

The Group×Age interaction was nonsignificant (*p* = 0.100); Levene’s *p* = 0.090, indicating no evidence of heteroscedasticity. The Shapiro–Wilk test for normality of the residuals for pH was not significant (*W* = 0.998, *p* = 0.999). Model residual diagnostics were acceptable.

ANCOVA showed a significant group effect (*F* = 16.55, *p* < 0.001, *η²p* = 0.398); age was not significant (*p* = 0.843). Age-adjusted EMMs (pH) were as follows: mid-cycle, 7.646; menstruating, 7.635; pregnant, 6.932; and menopausal, 6.633 (Table 2). Tukey post-hoc: mid-cycle and menstruating > pregnant and menopausal (most *p* < 0.001; mid-cycle vs. menopausal *p* = 0.010; menstruating vs. menopausal *p* = 0.023); mid-cycle vs. menstruating not significant (*p* = 0.999); and pregnant vs. menopausal not significant (*p* = 0.814) (Table 3).

### Oral hygiene scores

**Fig. 1:**
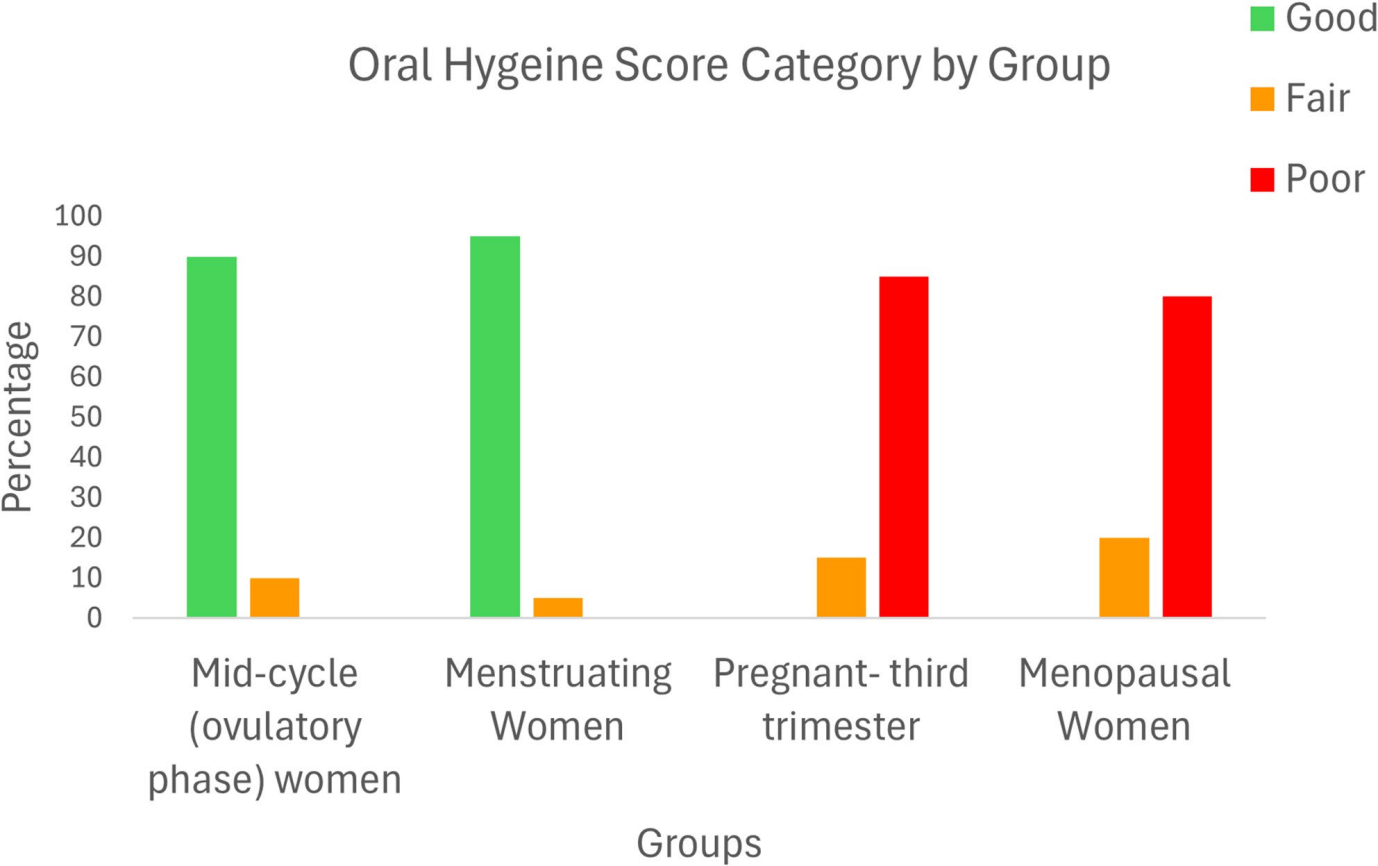
Distribution of Oral Hygiene Score Categories among Different Hormonal Groups of Women

Oral hygiene scores also varied significantly among the groups (Fisher’s exact test, *p* < 0.001). mid-cycle women and menstruating women predominantly fell into the “good” category for oral hygiene (90% and 95%, respectively). In contrast, pregnant and menopausal women exhibited poorer oral hygiene, with a substantial proportion categorized as “fair” or “poor.” Among pregnant women, 85% were classified as having “poor” oral hygiene, whereas 80% of menopausal women were similarly classified (Table 4; Fig. 1).

**Table 4:** Oral hygiene score category and participant group

	Oral Hygiene Score Category			
Group	**Good** **Freq. (%)**	**Fair** **Freq. (%)**	**Poor** **Freq. (%)**	**Total** **Freq. (%)**
Mid-cycle (ovulatory phase) women	18 (90)	2 (10)	0 (0)	20 (100)
Menstruating Women	19 (95)	1 (5)	0 (0)	20 (100)
Pregnant-third trimester	0 (0)	3 (15)	17 (85)	20 (100)
Menopausal Women	0 (0)	4 (20)	16(80)	20 (100)

Fisher’s exact test; *p* < 0.001

### Secondary finding

Exploratory analysis revealed a moderate positive correlation between the salivary flow rate and pH (*r* = 0.591, *p* < 0.001).

## Discussion

Women’s hormonal fluctuations at different life stages have a pronounced effect on their oral health. The hormonal milieu modulates the inflammatory response and vascular supply in oral tissues, making specific periods of a woman’s life (such as pregnancy and menopause) particularly challenging for oral health management. Elevated levels of estrogen and progesterone can lead to increased gingival vasodilation and capillary permeability, resulting in greater gingival exudate and inflammatory cell migration into tissues [18, 19]. This hormonal surge can also alter the oral microbiome and increase nutrient availability for pathogenic microbes, increasing the risk of gingival inflammation [19–21]. The present study provides clinical evidence of significant changes in saliva and oral hygiene across different hormonal phases. We observed that pregnant and postmenopausal women had significantly lower unstimulated salivary flow rates than women in the menstrual phase or control group did. This is in accordance with findings by Karnik et al. and Gill et al., who reported reduced salivary flow in both pregnant and menopausal women relative to nonpregnant controls [22, 23]. Similarly, Migliario et al. reported that pregnant women have lower salivary flow than nonpregnant women do​, which has been attributed to increased progesterone and estrogen levels during pregnancy [11]. One potential mechanism is the presence of hormone receptors in salivary gland tissue; elevated sex hormones may directly influence glandular function and suppress saliva secretion​ [1, 24]. Foglio-Bonda et al. reported that the salivary flow of menopausal women was significantly lower than that of premenopausal women​ [25]. The consistency of these findings indicates a strong association between hormonal changes (particularly high hormone levels during pregnancy and a low estrogen state during menopause) and diminished salivary gland activity.

The salivary flow rate is a critical determinant of salivary pH and buffering capacity. The similarity of the salivary flow rate and pH between menstruating and mid-cycle (ovulatory phase) women likely reflects their comparable hormonal profiles and age ranges, suggesting that physiological status rather than age is the dominant factor influencing these salivary parameters. In our study, the groups with reduced flow (pregnant and menopausal) also presented more acidic salivary pH levels. This aligns with the results of prior studies by Migliario et al. and Yilmaz et al., who reported that salivary pH tends to shift into an acidic range during pregnancy [10, 11]. The likely explanation is that a lower flow rate reduces the output of bicarbonate and other buffers in saliva, thereby decreasing the salivary pH and buffering capacity​. Our pairwise comparisons revealed that pregnant and menopausal women had significantly lower pH values than cycling women did. In contrast, between pregnant and menopausal women, the pH difference was insignificant, suggesting that any substantial deviation from the normal hormonal cycle, whether extremely high (pregnancy) or low (menopause) estrogen/progesterone, can negatively affect salivary alkalinity. Despite a wide age gap, this similarity suggests that the altered hormonal environments of excessive estrogen during pregnancy versus estrogen deficiency during menopause may exert similar suppressive effects on salivary gland function, suggesting that physiology rather than age is the primary driver.

Adequate salivary flow and a neutral pH protect oral tissues; when these conditions are compromised, the risk of dental caries and mucosal infections increases. The dramatically poorer oral hygiene scores observed in our pregnant and menopausal participants corroborate this concern. In our study, none of the pregnant or menopausal women had good oral hygiene; most had poor scores, whereas nearly all menstruating or middle-cycle women had good oral hygiene. Conversely, good oral hygiene among menstruating and mid-cycle (ovulatory phase) women could be attributed to hormonal stability and possibly better self-care habits often observed in younger individuals; however, balanced hormonal status likely contributes to better gingival resilience and oral cleanliness. This finding is in line with earlier reports connecting hormonal changes to periodontal health. Krejci et al. reported that fluctuations in female sex hormones can exacerbate the host response to plaque, potentially aggravating gingivitis and periodontitis [26]. More recent literature reinforces this link: menopause-related estrogen deficiency, for example, is associated with more significant periodontal bone loss and tooth mobility [5]. In pregnant women, increased gingival inflammation (pregnancy gingivitis) is standard. It can progress to periodontal disease if oral hygiene is neglected [27, 28]. One study noted significantly greater plaque and gingival indices in the prepartum period​ [3]. Our findings suggest that without special attention to oral care, pregnancy and menopause can predispose women to poor periodontal status and oral hygiene outcomes. Research has noted increased gingivitis and caries incidence during pregnancy, which is consistent with the poor hygiene and low pH environment we observed, which favor cariogenic activity [10, 29]. The reasons why pregnant and menopausal women fare worse in terms of oral health measures are worth discussing. In pregnancy, nausea, vomiting, dietary cravings, and fatigue may hinder regular oral hygiene practices, whereas hormonal gingival changes increase the sensitivity and susceptibility of gums to bleeding, possibly discouraging brushing​. Moreover, some pregnant women mistakenly avoid dental visits because of safety concerns, leading to untreated dental issues. During menopause, salivary gland hypofunction (often related to hormonal changes or age-related atrophy) can cause dry mouth, and the absence of estrogen may negatively affect collagen maintenance in the gingiva and bone density in the jaw​ [1, 5, 30]. Menopausal women might also be on medications (e.g., antihypertensives, antidepressants) that can further reduce saliva, compounding oral dryness. These factors can result in rapid plaque accumulation and periodontal deterioration if not managed with targeted oral hygiene interventions and possibly saliva substitutes or stimulants [5, 31, 32].

The results of our study underscore the need for integrative healthcare approaches. Obstetricians and gynecologists should advise pregnant and perimenopausal patients about maintaining oral hygiene and promptly refer them for dental care when issues arise. Likewise, dentists should be cognizant of the patient’s hormonal status; for example, scheduling nonurgent periodontal treatments outside the premenstrual phase might be beneficial for women who experience cyclical gingival flare-ups​. Importantly, improving oral health in pregnant women not only is crucial for the mother but also may benefit pregnancy outcomes, as severe periodontal disease has been linked to adverse outcomes such as preterm birth and low birth weight in some studies​.

The wide age range of the participants (22.78 to 55.78 years) reflects the natural distribution across the studied physiological groups. Although age-related changes in salivary gland function and oral health are well documented, several studies suggest that salivary parameters are more closely linked to hormonal influences than chronological age alone. For example, Krupa et al. reported that the duration of menopause significantly affects the salivary flow rate, highlighting the impact of hormonal changes on salivary function [33]. Similarly, Saluja et al. reported significantly lower salivary pH values in postmenopausal women, indicating that hormonal changes during menopause can affect salivary composition [15]. While hormonal fluctuations appear central to the differences observed, behavioral and lifestyle factors may also have played a role. Oral hygiene practices, dietary habits, frequency of dental visits, and access to preventive care can substantially influence salivary function and oral hygiene status. These unmeasured variables may partly explain the variation observed across groups and should be considered in future research alongside hormonal influences. The cross-sectional nature of our study limits the ability to fully disentangle age and hormonal effects, and future studies with age-matched cohorts or longitudinal follow-ups are recommended. Although dentition status (DMFT) and periodontal parameters (bleeding on probing, pocket depth, and clinical attachment loss) were recorded according to WHO criteria, these data were not the primary focus of the present analysis. The study was designed with the simplified oral hygiene index (OHI-S) as the main outcome to assess oral hygiene status across hormonal groups. Future studies with larger cohorts may explore these additional oral health indicators to provide a more comprehensive assessment.

Through exploratory correlation analysis, we observed a moderate positive association between the salivary flow rate and pH (*r* = 0.591, *p* < 0.001), suggesting that higher unstimulated flow corresponds with less acidic saliva. This aligns with physiological principles, as increased flow enhances bicarbonate buffering capacity. Prior studies also support this relationship: Schwerdt et al. demonstrated a correlation between salivary pH and flow in healthy volunteers, whereas Forcella et al. reported similar findings in children, with unstimulated salivary flow significantly correlating with pH and buffer capacity [34, 35]. Within our hormonal-phase groups, this correlation suggests that hormonal influences may jointly modulate flow and buffering capacity. It also remains unclear whether the hormonal effects on salivary flow and pH are reversible postpartum or with hormone replacement therapy in menopausal women, and future longitudinal studies should investigate this possibility.

This study has several limitations that should be acknowledged. The cross-sectional design precludes causal inference, and the inclusion of only third-trimester pregnant women limits generalizability to earlier stages of pregnancy. Methodological constraints include the use of unstimulated saliva and pH strips, which, while practical, are less precise than stimulated samples or digital pH meters are [17, 36, 37]. Future studies should consider incorporating both saliva types and more precise pH measurement methods to improve accuracy. In addition, unmeasured behavioral and contextual confounders-such as oral hygiene practices, dietary habits, socioeconomic status, psychosocial stress, and medication use may have influenced the outcomes. In addition, subjective symptoms such as xerostomia or oral discomfort were not assessed, which could have provided complementary information to the objective salivary measures. Although we attempted to minimize confounding by excluding systemic illnesses and performing ANCOVA with age as a covariate, residual confounding cannot be excluded. Despite these limitations, the consistency of our findings with those of prior studies suggests that the observed associations reflect the genuine physiological influences of hormonal fluctuations on salivary parameters and oral health.

## Conclusion

Hormonal changes during different stages of a woman’s life, especially in the third trimester of pregnancy and during menopause, are associated with reduced salivary flow, lower pH, and poorer oral hygiene. These findings highlight the importance of considering hormonal status in preventive oral health care and suggest that targeted strategies are needed to reduce oral disease risk in hormonally sensitive periods. Interdisciplinary collaboration between gynecologists and dental professionals may play a crucial role in promoting women’s overall health. Future longitudinal studies including all stages of pregnancy and accounting for behavioral confounders are warranted to strengthen causal inference.

## Data Availability

The datasets used and/or analyzed during the current study are available from the corresponding author on reasonable request.
